# Supplementary material for: Prevalence and Impact of Vitamin D Deficiency in Critically Ill Cancer Patients Admitted to the Intensive Care Unit
Source: Nutrients. 2020 Dec 23;13(1):22. doi: 10.3390/nu13010022 (PMC7822404; doi:10.3390/nu13010022)
Supplement: Supplementary file 1 [file nutrients-13-00022-s001.pdf]

nutrients

Supplemental Material

Prevalence and impact of Vitamin D deficiency in critically ill cancer patients admitted to the intensive care unit

Table S1: Characteristics of patients from the registry included and not included in the study

	Study patients (n=178)	Non-study patients (n=163)
Sex, male	117 (66)	115 (71)
Age, years	61 (53-71)	64 (56-71)
Body mass index (kg/m^2)	25.2 (21.9-29.1)	25.1 (23-29.2)
Type of cancer		
Hematologic	108 (61)	97 (59)
Solid cancer	70 (39)	66 (41)
Disease state		
Naïve	34 (19)	31 (19)
Complete or partial remission	67 (38)	56 (35)
Progression/Relapsed/Refractory	37 (21)	38 (23)
Undetermined/Unknown	40 (22)	38 (24)
Time from diagnosis, months	15.4 (2.6-44)	12.6 (3.7-38.3)
Time since last therapy, months	1 (0.3-16)	1.4 (0.3-17.7)
Allogeneic HSCT	25 (14)	18 (11)
Charlson Comorbidity Index (CCI)	3 (2-5)	4 (2-6)
Hospital to ICU admission, days	3 (0-13)	4 (0-14)
Reasons for ICU admission		
Acute respiratory failure	121 (68)	113 (69)
Infection	107 (60)	107 (66)
Shock	87 (49)	82 (50)
Acute kidney injury	73 (41)	75 (46)
Neurological dysfunction	22 (12)	24 (15)
SAPS II at ICU admission	52 (40-66)	53 (43-73)
SOFA score at ICU admission	10 (7-13)	10 (6-13)
Life-supporting interventions		
Vasopressors	135 (76)	129 (80)
Invasive ventilation	109 (61)	103 (64)
Renal replacement therapy	44 (25)	42 (26)
Extracorporeal life support	15 (8)	14 (9)
ICU survival	90 (63)*	91 (56)
Hospital survival	71 (49)*	74 (45)

*Data analyzed for 144 patients (see Methods section). Abbreviations: HSCT = hematopoietic stem cell transplantation; ICU = intensive care unit; SAPS II = Simplified Acute Physiologic Score II; SOFA = Sepsis-Related Organ Failure Assessment Score.

Table S2: Cancer diagnoses in the study population

Cancer diagnosis	n=178
Hematologic	
Non-Hodgkin lymphoma	36 (20)
Hodgkin's lymphoma	5 (3)
Acute myeloid leukemia	37 (21)
Acute lymphoblastic leukemia	5 (3)
Myeloma	10 (6)
Chronic myeloid leukemia	3 (2)
Chronic lymphocytic leukemia	4 (2)
Myelodysplastic syndrome	5 (3)
Other	3 (2)
Solid cancer	
Lung	13 (7)
Head and neck	11 (6)
Breast	6 (3)
Prostate	4 (2)
Upper gastrointestinal tract	6 (3)
Liver/Bile/Pancreas	7 (4)
Colorectal	3 (2)
Urologic/Gynecologic	10 (6)
Melanoma	2 (1)
Sarcoma/Soft tissue	6 (3)
Other	2 (1)

Data are presented as absolute numbers (%).

Table S3: Inter-group comparisons according to 25(OH) D deficiency (<20 ng/mL) and severe deficiency (≤12 ng/mL)

	**25(OH) D ≥20 ng/mL (n=47)**	**25(OH) D <20 ng/mL (n=131)**	**25(OH) D >12 ng/mL (n=82)**	**25(OH) D ≤12 ng/mL (n=96)**
Sex, male	28 (60)	89 (68)	53 (65)	64 (67)
Age, years	**65 (55-73)**	**59 (50-69)***	63 (52-71)	60 (54-69)
Body mass index (kg/m^2)	24.5 (20.8-28.5)	25.3 (22-29.2)	25.9 (21.6-28.5)	24.8 (22-30)
Season of ICU admission				
Spring	14 (30)	39 (30)	22 (27)	31 (32)
Summer	9 (19)	36 (28)	19 (23)	26 (27)
Autumn	14 (30)	28 (21)	24 (29)	18 (19)
Winter	10 (21)	28 (21)	17 (21)	21 (22)
Type of cancer				
Hematologic	26 (55)	82 (63)	52 (63)	56 (58)
Solid cancer	21 (45)	49 (37)	30 (37)	40 (42)
Disease state				
Naïve	11 (23)	23 (18)	14 (17)	20 (21)
Complete or partial remission	16 (34)	51 (39)	31 (38)	36 (37)
Relapsed or refractory	**4 (9)**	**33 (25)***	16 (19)	21 (22)
Undetermined/Unknown	16 (34)	24 (18)	21 (26)	19 (20)
Time from diagnosis, months	16 (1.9-54.4)	14.7 (2.7-37.9)	14.7 (2.6-47.3)	16.7 (2.6-38.4)
Time since last therapy, months	1.6 (0.3-45.2)	1 (0.2-7.8)[%]	1.1 (0.3-30.7)	0.9 (0.2-6.3)[%]
Therapy during last 12 months	**21 (45)**	**85 (65)***	45 (55)	61 (64)
Allogeneic HSCT	5 (11)	20 (15)	10 (12)	15 (16)
Charlson Comorbidity Index (CCI)	3 (2-5)	3 (2-5)	3 (2-5)	3 (2-5)
Hospital to ICU admission, days	2 (0-7)	4 (0-16)[§]	3 (0-13)	3 (0-15)
Reasons for ICU admission				
Acute respiratory failure	34 (72)	87 (66)	58 (71)	63 (66)
Infection	23 (49)	84 (64)[§]	47 (57)	60 (63)
Shock	21 (45)	66 (50)	40 (49)	47 (49)
Acute kidney injury	15 (32)	58 (44)[%]	32 (39)	41 (43)
Neurological dysfunction	4 (9)	18 (14)	8 (10)	14 (15)
SAPSII at ICU admission	48 (43-58)	54 (39-68)	48 (40-62)	55 (40-67)
SOFA score at ICU admission	**9 (7-10)**	**11 (8-14)***	9 (7-12)	11 (8-13)[§]
Life-supporting interventions				
Vasopressors	35 (75)	100 (76)	62 (76)	73 (76)
Invasive ventilation	25 (53)	84 (64)	51 (62)	58 (60)
Renal replacement therapy	7 (15)	37 (28)[§]	16 (20)	28 (29)[%]
Extracorporeal life support	3 (6)	12 (9)	6 (7)	9 (9)
25(OH)-D_3 (ng/mL)	**28.4 (24-31.8)**	**9.4 (6.2-12.2)***	**22.7 (17-29.4)**	**7.8 (5.4-9.9)***
25(OH)-D_3 <20 ng/mL	0	131 (100)	35 (43)	96 (100)
25(OH)-D_3 ≤12 ng/mL	0	96 (73)	0	96 (100)
Total calcium (mmol/L)	2.02 (1.91-2.12)	2.0 (1.87-2.09)	2.04 (1.90-2.12)	1.95 (1.86-2.08)[%]
Ionized calcium (mmol/L)	1.09 (1.05-1.14)	1.12 (1.04-1.20)[%]	1.1 (1.05-1.18)	1.1 (1.02-1.17)
Phosphate (mmol/L)	1.23 (0.9-1.42)	1.24 (0.88-1.68)	1.24 (0.89-1.48)	1.24 (0.87-1.71)
iPTH (pg/mL)	52.7 (32.4-113.4)	50.1 (29.5-119.2)	47.2 (29.3-99.3)	55.5 (30.8-140.3)
WBC (G/L)	12.8 (6.6-19.2)	10.9 (2.8-17.5)	12.2 (3.1-18.7)	12.2 (2.8-17.1)
Albumin (mg/dL)	25 (22.1-28.2)	26.1 (23.1-30.3)	25.7 (23.3-29.3)	25.7 (22.8-30.2)
Creatinine (mg/dL)	1 (0.8-1.67)	1.3 (0.79-2.44)	1.1 (0.8-2)	1.37 (0.75.2.48)
CRP (mg/dL)	18.4 (6.2-40.8)	21.4 (7.5-34.1)	18.9 (6-40)	23.3 (7.6-33.4)

Data are presented as absolute numbers (%) or median (interquartile range). Abbreviations: CRP = C-reactive protein; G/L = Giga per liters; HSCT = hematopoietic stem cell transplantation; ICU = intensive care unit; iPTH = intact parathyroid hormone; SAPS II = Simplified Acute Physiologic Score II; SOFA = Sepsis-Related Organ Failure Assessment Score; WBC = white blood cell count; P-values are indicated by superscript signs: * = $p<0.05$ (bold), § = $p\geq0.05$ and ≤0.10, % = $p>0.10$ and ≤0.20.

Table S4: Comparison of ICU survivors and non-survivors

	ICU survivors (n=90)	ICU non-survivors (n=54)	*p*
Sex, male	61 (68)	36 (67)	1.0
Age, years	59 (51-69)	63 (56-71)	0.16
Body mass index (kg/m²)	24.7 (21.2-27.7)	26.1 (22.8-30.3)	0.05
Type of malignancy			0.48
Hematologic	52 (58)	35 (65)	
Solid cancer	38 (42)	19 (35)	
Disease state			0.31
Naïve	13 (14)	10 (19)	
Complete or partial remission	41 (46)	19 (35)	
Relapsed or refractory	15 (17)	15 (28)	
Undetermined/Unknown	21 (23)	10 (19)	
Time from diagnosis, months	20.2 (2.8-54.4)	11.7 (2.7-35.7)	0.57
Time since last therapy, months	1 (0.3-21.2)	0.9 (0.2-5.6)	0.36
Therapy during last 12 months	54 (60)	36 (67)	0.48
Allogeneic HSCT	11 (12)	10 (19)	0.34
Charlson Comorbidity Index (CCI)	3 (2-4)	3 (2-5)	0.64
Hospital to ICU admission, days	3 (0-16)	3 (1-11)	0.96
Reasons for ICU admission			
Acute respiratory failure	54 (60)	36 (67)	0.48
Infection	44 (49)	40 (74)	**<0.01**
Shock	44 (49)	27 (50)	1.0
Acute kidney injury	28 (31)	26 (48)	0.05
Neurological dysfunction	8 (9)	8 (15)	0.29
SAPSII at ICU admission	9 (7-12)	11 (8-15)	**0.02**
SOFA score at ICU admission	9 (7-12)	11 (8-15)	**<0.01**
Life-supporting interventions			
Vasopressors	58 (64)	51 (94)	**<0.01**
Invasive ventilation	42 (47)	45 (83)	**<0.01**
Renal replacement therapy	11 (12)	23 (43)	**<0.01**
Extracorporeal life support	5 (6)	7 (13)	0.13
25(OH)-D_3 (ng/mL)	12.5 (7.4-23.3)	10.2 (6.4-16.5)	0.12
25(OH)-D_3 <20 ng/mL	62 (69)	44 (82)	0.12
25(OH)-D_3 ≤12 ng/mL	44 (49)	34 (63)	0.12
Length of ICU stay, days	10 (4-19)	11 (4-23)	0.96
Hospital survival	71 (79)	0	
1-year survival*	45 (52)	0	

*Data analyzed for 140 patients. Data are presented as absolute numbers (%) or median (interquartile range). Abbreviations: HSCT = hematopoietic stem cell transplantation; ICU = intensive care unit; SAPS II = Simplified Acute Physiologic Score II; SOFA = Sepsis-Related Organ Failure Assessment Score; P-values <0.05 are highlighted.

Table S5: Comparison of hospital survivors and non-survivors

	Hospital survivors (n=71)	Hospital non-survivors (n=73)	*p*
Sex, male	50 (70)	47 (64)	0.48
Age, years	59 (52-68)	63 (54-72)	0.16
Body mass index (kg/m^2)	24.7 (21.3-27.4)	25.7 (22.1-30.1)	0.16
Type of malignancy			0.61
Hematologic	41 (58)	46 (63)	
Solid cancer	30 (42)	27 (37)	
Disease state			**<0.01**
Naïve	11 (16)	12 (16)	
Complete or partial remission	39 (55)	21 (29)	
Progression/Relapsed/Refractory	8 (11)	22 (30)	
Undetermined/Unknown	13 (18)	18 (25)	
Time from diagnosis, months	21.4 (3.4-55.2)	11.6 (2.6-35)	0.27
Time since last therapy, months	1.1 (0.3-26.7)	0.9 (0.1-6.7)	0.16
Therapy during last 12 months	40 (56)	50 (69)	0.17
Allogeneic HSCT	9 (13)	12 (16)	0.64
Charlson Comorbidity Index (CCI)	3 (2-4)	3 (2-5)	0.63
Hospital to ICU admission, days	3 (0-16)	4 (1-15)	0.76
Reasons for ICU admission			
Respiratory failure	44 (62)	46 (63)	1.0
Infection	34 (48)	50 (69)	**0.02**
Shock	38 (54)	33 (45)	0.41
Acute kidney injury	23 (32)	31 (43)	0.23
Neurological dysfunction	4 (6)	12 (16)	0.06
SAPSII at ICU admission	48 (37-62)	55 (44-67)	0.10
SOFA score at ICU admission	9 (8-12)	11 (8-13)	0.12
Life supporting interventions			
Vasopressors	45 (63)	64 (88)	**<0.01**
Invasive ventilation	29 (41)	58 (80)	**<0.01**
Renal replacement therapy	7 (10)	27 (37)	**<0.01**
Extracorporeal life support	4 (6)	8 (11)	0.37
25(OH)-D_3 (ng/mL)	13.4 (6.8-26.1)	10.4 (6.9-16.5)	0.10
25(OH)-D_3 <20 ng/mL	46 (65)	60 (82)	**0.02**
25(OH)-D_3 ≤12 ng/mL	32 (45)	46 (63)	**0.04**
Length of ICU stay, days	9 (4-15)	11 (4-23)	0.38
ICU survival	71 (100)	19 (26)	
1-year survival*	45 (67)	0	

*Data analyzed for 140 patients. Data are presented as absolute numbers (%) or median (interquartile range). Abbreviations: HSCT = hematopoietic stem cell transplantation; ICU = intensive care unit; SAPS II = Simplified Acute Physiologic Score II; SOFA = Sepsis-Related Organ Failure Assessment Score; P-values <0.05 are highlighted.

Table S6: Comparison of 1-year survivors and non-survivors

	1-year survivors (n=45)	1-year non-survivors (n=95)	*p*
Sex, male	28 (62)	66 (70)	0.44
Age, years	58 (51-69)	62 (54-71)	0.15
Body mass index (kg/m²)	25.1 (21-27.1)	25 (22-30.1)	0.19
Type of malignancy			0.58
Hematologic	26 (58)	60 (63)	
Solid cancer	19 (42)	35 (37)	
Disease state			**<0.01**
Naïve	8 (18)	15 (16)	
Complete or partial remission	27 (60)	30 (32)	
Progression/Relapsed/Refractory	3 (7)	27 (28)	
Undetermined/Unknown	7 (16)	23 (24)	
Time from diagnosis, months	10.7 (3.1-61.8)	11.7 (2.7-34.2)	0.20
Time since last therapy, months	0.7 (0.4-29.8)	0.9 (0.1-6.8)	0.21
Therapy during last 12 months	25 (56)	64 (67)	0.19
Allogeneic HSCT	5 (11)	16 (17)	0.45
Charlson Comorbidity Index (CCI)	3 (2-4)	3 (2-5)	0.75
Hospital to ICU admission, days	1 (0-17)	3 (1-12)	0.54
Reasons for ICU admission			
Respiratory failure	30 (67)	59 (62)	0.71
Infection	18 (40)	64 (67)	**<0.01**
Shock	23 (51)	46 (48)	0.86
Acute kidney injury	16 (36)	37 (39)	0.85
Neurological dysfunction	2 (4)	14 (15)	0.09
SAPSII at ICU admission	48 (36-61)	52 (41-67)	0.18
SOFA score at ICU admission	9 (7-12)	11 (8-13)	0.06
Life-supporting interventions			
Vasopressors	27 (69)	79 (83)	**<0.01**
Invasive ventilation	19 (42)	66 (70)	**<0.01**
Renal replacement therapy	6 (13)	28 (30)	0.06
Extracorporeal life support	4 (9)	8 (8)	1.0
25(OH)-D_3 (ng/mL)	17.6 (8.9-26.2)	10.4 (6.7-18.5)	**0.03**
25(OH)-D_3 <20 ng/mL	27 (60)	76 (80)	**0.02**
25(OH)-D_3 ≤12 ng/mL	16 (36)	59 (62)	**<0.01**
Length of ICU stay, days	11 (4-16)	9 (4-22)	
ICU survival	45 (100)	41 (43)	
Hospital survival	45 (100)	22 (23)	

Data are presented as absolute numbers (%) or median (interquartile range). Abbreviations: HSCT = hematopoietic stem cell transplantation; ICU = intensive care unit; SAPS II = Simplified Acute Physiologic Score II; SOFA = Sepsis-Related Organ Failure Assessment Score; P-values <0.05 are highlighted.

Table S7: Comparison of 1-year survivors and non-survivors among ICU survivors

	1-year survivors (n=45)	1-year non-survivors (n=41)	*p*
Sex, male	28 (62)	30 (73)	0.36
Age, years	58 (51-69)	59 (51-70)	0.47
Body mass index (kg/m^2)	25.1 (21-27.1)	24.1 (21.3-29.9)	0.84
Type of malignancy			0.83
Hematologic	26 (58)	25 (61)	
Solid cancer	19 (42)	16 (39)	
Disease state			**<0.01**
Naïve	8 (18)	5 (12)	
Complete or partial remission	27 (60)	11 (27)	
Progression/Relapsed/Refractory	3 (7)	12 (29)	
Undetermined/Unknown	7 (16)	13 (32)	
Time from diagnosis, months	10.7 (3.1-61.8)	12 (2.6-30.7)	0.25
Time since last therapy, months	0.7 (0.4-29.8)	0.9 (0.1-10.4)	0.34
Therapy during last 12 months	25 (56)	28 (68)	0.27
Allogeneic HSCT	5 (11)	6 (15)	0.75
Charlson Comorbidity Index (CCI)	3 (2-4)	3 (2-6)	0.46
Hospital to ICU admission, days	1 (0-17)	5 (0-14)	0.68
Reasons for ICU admission			
Acute respiratory failure	30 (67)	23 (56)	0.38
Infection	18 (40)	24 (59)	0.13
Shock	23 (51)	19 (46)	0.67
Acute kidney injury	16 (36)	11 (27)	0.49
Neurological dysfunction	2 (4)	6 (15)	0.14
SAPSII at ICU admission	48 (36-61)	47 (40-64)	0.94
SOFA score at ICU admission	9 (7-12)	9 (8-13)	0.59
Life-supporting interventions			
Vasopressors	27 (69)	28 (68)	0.50
Invasive ventilation	19 (42)	21 (51)	0.52
Renal replacement therapy	6 (13)	5 (12)	1.0
Extracorporeal life support	4 (9)	1 (2)	0.36
25(OH)-D_3 (ng/mL)	17.6 (8.9-26.2)	10.9 (6.8-19.7)	0.15
25(OH)-D_3 <20 ng/mL	27 (60)	32 (78)	0.10
25(OH)-D_3 ≤12 ng/mL	16 (36)	25 (61)	**0.03**
Length of ICU stay, days	11 (4-16)	7 (4-22)	0.68
Hospital survival	45 (100)	22 (54)	

Data are presented as absolute numbers (%) or median (interquartile range). Abbreviations: HSCT = hematopoietic stem cell transplantation; ICU = intensive care unit; SAPS II = Simplified Acute Physiologic Score II; SOFA = Sepsis-Related Organ Failure Assessment Score; P-values <0.05 are highlighted.
